# Can Robotic Systems Promote Self-Disclosure in Adolescents with Autism Spectrum Disorder? A Pilot Study

**DOI:** 10.3389/fpsyt.2018.00036

**Published:** 2018-02-09

**Authors:** Hirokazu Kumazaki, Zachary Warren, Amy Swanson, Yuichiro Yoshikawa, Yoshio Matsumoto, Hideyuki Takahashi, Nilanjan Sarkar, Hiroshi Ishiguro, Masaru Mimura, Yoshio Minabe, Mitsuru Kikuchi

**Affiliations:** ^1^Department of Clinical Research on Social Recognition and Memory, Research Center for Child Mental Development, Kanazawa University, Ishikawa, Japan; ^2^Department of Pediatrics, Vanderbilt Kennedy Center, Nashville, TN, United States; ^3^Department of Psychiatry, Vanderbilt Kennedy Center, Nashville, TN, United States; ^4^Departments of Special Education, Vanderbilt Kennedy Center, Nashville, TN, United States; ^5^Treatment and Research Institute of Autism Spectrum Disorders, Vanderbilt Kennedy Center, Nashville, TN, United States; ^6^Department of Systems Innovation, Graduate School of Engineering Science, Osaka University, Osaka, Japan; ^7^Interaction Service Robotics Research Group, Intelligent Systems Institute, National Institute of Advanced Industrial Science and Technology, Ibaraki, Japan; ^8^Department of Mechanical Engineering, Vanderbilt University, Nashville, TN, United States; ^9^Department of Neuropsychiatry, Keio University School of Medicine, Tokyo, Japan

**Keywords:** autism spectrum disorder, self-disclosure, robotics, android robot, simplistic humanoid

## Abstract

Research suggests that many individuals with autism spectrum disorder (ASD) often demonstrate challenges providing appropriate levels of information during conversational interchanges. Considering the preference of individuals with ASD, and recent rapid technological advances, robotic systems may yield promise in promoting certain aspects of conversation and interaction such as self-disclosure of appropriate personal information. In the current work, we evaluated personal disclosures of events with specific emotional content across two differing robotic systems (android and simplistic humanoid) and human interactions. Nineteen participants were enrolled in this study: 11 (2 women and 9 men) adolescents with ASD and 8 (4 women and 4 men) adolescents with TD. Each participant completed a sequence of three interactions in a random order. Results indicated differences regarding comfort level and length of disclosures between adolescents with ASD and typically developing (TD) controls in relation to system interactions. Specifically, adolescents with ASD showed a preference for interacting with the robotic systems compared to TD controls and demonstrated lengthier disclosures when interacting with the visually simple humanoid robot compared to interacting with human interviewer. The findings suggest that robotic systems may be useful in eliciting and promoting aspects of social communication such as self-disclosure for some individuals with ASD.

## Introduction

Individuals with autism spectrum disorder (ASD) display impairments in social communication and interaction, often including challenges related to appropriate engagement in conversation. Specific challenges may be related to differences in narrative competence and theory of mind including differences in knowing what information is appropriate to disclose to others and how to successfully disclose information (1). Self-disclosure is the process by which people reveal personal information about themselves to others and is important in all types and stages of social relationships (2). Reciprocity *via* effective self-disclosure can lead to positive outcomes in initial interactions and promote further disclosure and relationship building (3). Individuals with ASD sometimes hesitate to disclose information to others due to challenges in understanding such interchanges or recognizing the potential value in relational reciprocity, as well as differences in social motivation (1). Previous studies have consistently demonstrated that individuals with ASD provide fewer and shorter self-disclosure statements in personal narratives when compared with individuals with typical development (4–7).

Recent rapid technological advances have enabled robots to fulfill a variety of human-like functions, leading researchers to use such technology for the development and subsequent validation of robotic interventions for individuals with ASD (8, 9). Given that social communication intervention approaches may be most effective when individuals with ASD are engaged in motivating activities and settings (10) and a growing body of literature suggests intrinsic motivation during interaction with robotic and technological systems (11–17), deploying such systems in meaningful interventions settings may represent a potential use of such technology.

Growing anecdotal evidence indicates that the use of robots may provide unique opportunities for assisting individuals with ASD (18–21). For example, Kaboski et al. (20) reported a novel intervention using humanoid robots to reduce social anxiety and improve social and vocational skills for adolescents with ASD. Zheng et al. (21) presented a humanoid robot to draw attention from children with ASD and capitalized on the increased attention to generate opportunities to teach gestures more effectively compared with a human therapist. In order to better understand whether robotic systems might be helpful in promoting self-disclosure for individuals with ASD, we designed and tested a controlled interaction paradigm comparing different robotic systems (i.e., visually simple robot and android robot) in relation to controlled human interaction.

We compared the difference of impression (i.e., reported preference) in communicating with two types of humanoid robots and human interviewer, and ratio of change in measured length of disclosure statements in order to examine potential differences between adolescents with ASD and typically developing (TD) controls. We hypothesized that adolescents with ASD would report a greater preference for communicating with the robotic systems than TD adolescents, and demonstrate lengthier disclosures within the paradigm.

## Materials and Methods

### Participants

The current study was approved by the ethics committee of the Vanderbilt University. All procedures involving human participants were conducted in accordance with the ethical standards of the institutional and/or national research committee and with the 1964 Helsinki Declaration and its later amendments or comparable ethical standards. All participants were recruited through existing university-based registries. After a complete explanation of the study, all the participants provided written informed consent. All participants agreed to participate in the study. Eleven participants with ASD (age m = 15.91; SD = 1.20) and eight participants with TD (age m = 15.73; SD = 1.57) completed the study. All adolescents with ASD had received a clinical diagnosis of ASD based on DSM-5 criteria (22) from a licensed clinical psychologist and scored at or above the clinical cutoff on the Autism Diagnostic Observation Schedule, Second Edition (ADOS-2) (23). Estimates of cognitive functioning for both groups were available from the existing registry on the Stanford–Binet, Fifth Edition (SB-5) (24). All parents in both groups also completed both the Social Communication Questionnaire (SCQ) (25) and the Social Responsiveness Scale, Second Edition (SRS-2) (26) to screen for clinically significant ASD symptoms in the TD group and to index of current symptoms in the ASD group.

### Robotic Systems

**Figure 1:**
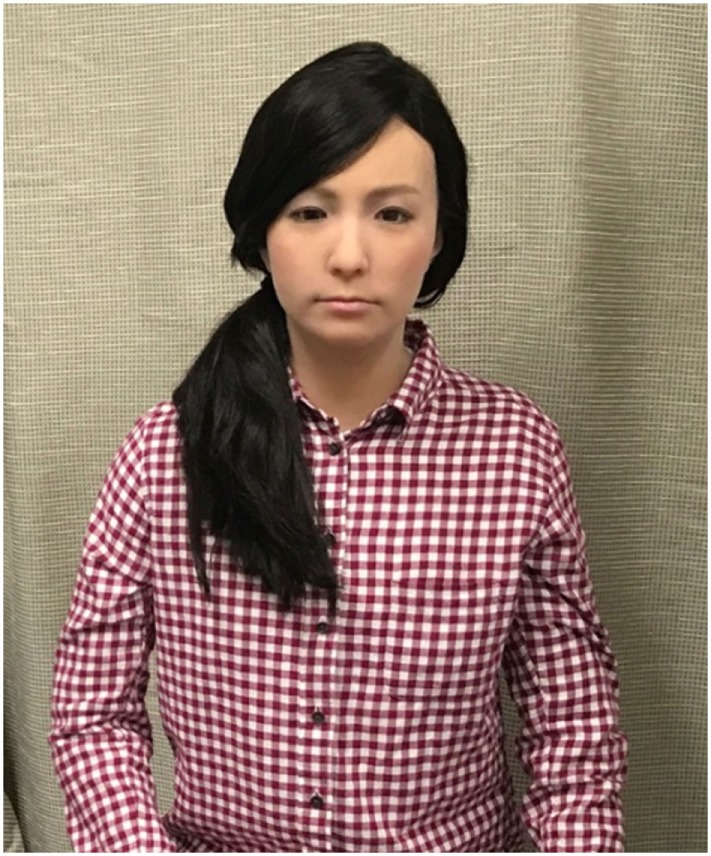
ACTROID-F (android robot).

**Figure 2:**
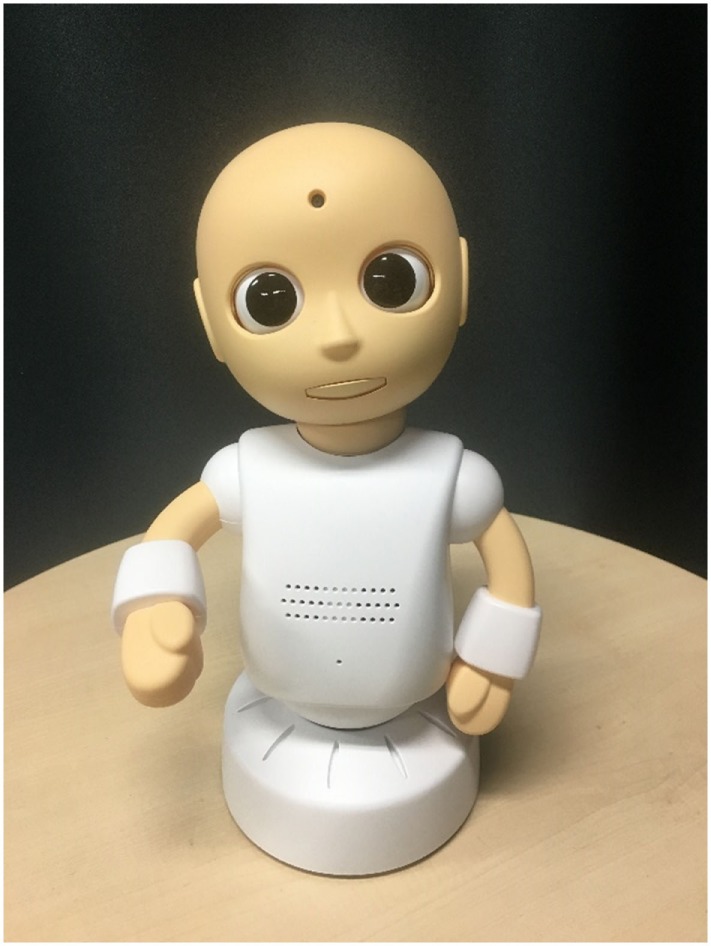
CommU (visually simple robot).

Both robots were tele-operated to engage in confederate and protocol controlled semi-structured conversations with participants. To elicit the belief that the robots were behaving and reacting autonomously, we adopted a remote control system similar to those conventionally used in robotics research (27). The Android robot employed was ACTROID-F (Kokoro Co., Ltd.), a female version of android robot with an appearance similar to that of a real person (see Figure 1) (28, 29) (i.e., its body is designed to have remarkably similar proportions, facial features, hair texture, and hairstyle to that of a human). ACTROID-F has been previously used to conduct experiments primarily designed to help adolescents with ASD participate effectively in conversational tasks (e.g., job interviews, conversations, and social skills interventions). The visually simple robot used in this study was a CommU (Vstone Co., Ltd.) (see Figure 2) (30). It is about 0.3-m tall and has a limited number of body parts consisting of head, torso, waist, and two arms, which are apparently less humanlike, although they are still expected to evoke humanness. The distinguishing feature of the robot is the high degree of freedom of the eyes, which can represent its attention.

### Procedure

**Figure 3:**
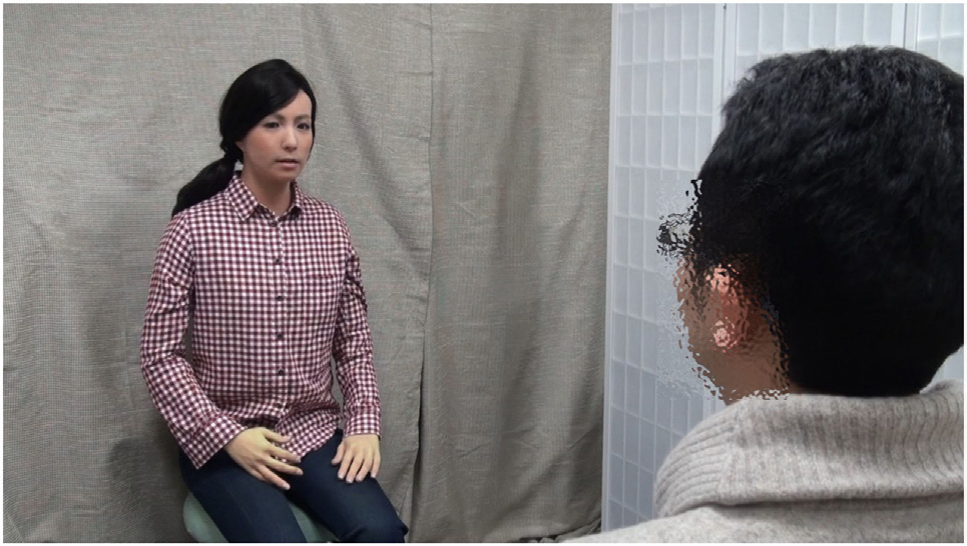
Typical interaction with a robot.

Each participant completed a sequence of three interaction conditions in random order, all of which were guided and took place in a standard clinical assessment room. We ensured a balance in the order of conditions to a certain extent. Please refer to Supplementary Materials for this order. Figure 3 provides an example of how participants typically interacted with the robots. The person in Figure 3 has given written and informed consent to publish this image. Prior to each session, both robots and human interviewer were situated in individual booths divided by opaque room dividers. We involved a variety of human interviewers (e.g., 25-year-old Caucasian woman). The two robots were operated by researchers seated in front of a terminal computer located in an adjacent observation room separated by a one-way mirror so that they were not visible during the session. Each trial lasted as long as the participants chose to converse around the presented topic and ended when the participant answered the question, or communicated that he/she did not wish to answer the question or discuss the topic. The average duration of each trial was approximately 5 min. The human interviewer and two robots followed a specific interview script and protocol to elicit self-disclosure on events or feelings across all interviews. The scripts followed the same basic structure. Specifically, participants were asked to share the happiest, saddest, and most embarrassing thing that happened to them at home, school, or outside of school. Please refer to Supplementary Material for examples of scripts.

At the close of the session, participants completed a self-report survey using a 9-point Likert scale to rank their level of enjoyment, embarrassment, stress, and boredom while communicating with each agent. Using audio recordings collected during the experimental session, the research team transcribed and totaled the number of words used in each exchange between participant and agent across conditions.

### Data Analysis

We performed statistical analysis using SPSS version 24.0 (IBM, Armonk, NY, USA). The differences of age, IQ, SRS-2, and SCQ score between the groups were analyzed using an independent samples *t*-test. The difference in gender proportion was analyzed using the χ^2^-test. Differences in the self-reported preference ratings communicating with the two robots between individuals with ASD and TD was analyzed using the Mann–Whitney *U*-test, as well as the ratio of number of words used in self-disclosure between interactions with each robot vs. human interviewer. As a complimentary analysis, to ascertain whether one or both robots promote more self-disclosure compared to a human interviewer, we compared the number of words in self-disclosure between each robot and human using a Wilcoxon signed-rank test. Spearman’s rank correlation coefficients were used to explore the relationships between IQ and the number of words in self-disclosure to each robot and human. An alpha level of 0.05 was used for these analyses.

## Results

In total, 11 adolescents with ASD and 8 TD adolescents took part in the study. All participants completed the experimental procedure and the semi-structured interviews. No significant differences were found between ASD and TD groups with regards to mean age (*p* = 0.77), gender proportion (*p* = 0.33) and IQ (*p* = 0.08). As expected, there were significant differences with regard to SRS-2 (*p* < 0.01) and SCQ (*p* < 0.01) between groups of adolescents with ASD and TD. Details are presented in Table 1.

**Table 1:** Participant characteristics.

Characteristics	ASD (*n* = 11), mean (SD)	TD (*n* = 8), mean (SD)	Statistics, *t*or χ^2^	*df*	*p*
Age in years	15.91 (1.20)	15.73 (1.57)	*t* = −0.289	17	0.78
Sex (male:female)	9:2	4:4	χ^2^ = 2.17	1	0.33
IQ	96.7 (18.5)	112.4 (17.3)	*t* = 1.871	17	0.08
SRS-2	73.4 (11.6)	47.6 (8.5)	*t* = −5.330	17	<0.01
SCQ	2.9 (2.7)	20.8 (7.8)	*t* = −6.205	17	<0.01

*C-H-A ASD, autism spectrum disorder; TD, typically developing; SRS-2, Social Responsiveness Scale—Second Edition T-Score; SCQ, Social Communication Questionnaire Lifetime Total Score*.

Significant differences in reported levels of enjoyment while communicating with the android robot were found between adolescents with ASD (5.82 ± 0.59; mean ± SEM) and TD controls (3.50 ± 0.68) (*p* < 0.05), as well as for the visually simple robot ASD: (6.36 ± 0.72); TD: (4.00 ± 0.71) (*p* < 0.05). There were no differences between ASD and TD participants in reported levels of other emotions: embarrassment, stress, and boredom while communicating with the two robots. Details are presented in Table 2.

**Table 2:** Overall impressions of communicating with each robot.

Group	ASD (*n* = 11)			TD (*n* = 8)			Mann–Whitney test	
	Q_1_	Median	Q_3_	Q_1_	Median	Q_3_	*U*	*p*
**Android robot**								
Feel enjoyable	4	5	8	2	3.5	4.75	16.50	0.02*
Feel embarrassed	3	5	7	2.5	5	7.5	42.00	0.90
Feel stressed	1	3	5	2.25	3.5	6	36.50	0.55
Feel bored	1	2	3	2	3	5.75	27.00	0.18

**Visually simple robot**								
Feel enjoyable	5	6	9	2	5	5.75	17.50	0.03*
Feel embarrassed	1	4	6	1	3	3	31.50	0.31
Feel stressed	1	3	5	1	2.5	3.75	39.00	0.72
Feel bored	1	2	4	1.25	3	3.75	35.00	0.18

*ASD, autism spectrum disorder; TD, typically developing; Q_1_, first quartile; Q_3_, third quartile*.

*Significant at p < 0.05.

Significant differences were observed between adolescents with ASD and TD adolescents in the ratio of the number of words used in self-disclosure between interactions with the visually simple robot vs. the human interviewer regarding the happiest experiences (ASD: 178.25 ± 39.68%; TD: 95.45 ± 31.74%), the most embarrassing experiences (ASD: 249.98 ± 46.04%; TD: 87.91 ± 26.09%), and all experiences (ASD: 176.30 ± 28.21%; TD: 85.62 ± 24.21%). There were no significant differences in the ratio of the number of words used in self-disclosure between interactions with the android robot vs. the human interviewer regarding any content or the total content between adolescents with ASD and TD controls. The details are presented in Table 3.

**Table 3:** Ratio of number of words used in self-disclosure between interactions with each robot vs. human interviewer.

Contents of speech	ASD (*n* = 11)			TD (*n* = 8)			Mann–Whitney test	
	Q_1_	Median	Q_3_	Q_1_	Median	Q_3_	*U*	*p*
**Android robot/human**								
The happiest thing	0.85	1.00	1.75	0.46	1.11	1.80	36.00	0.55
The saddest thing	0.58	1.33	1.75	0.56	0.80	1.35	31.00	0.31
The most embarrassment thing	0.60	0.88	1.50	0.49	0.57	0.90	27.50	0.18

Total	0.80	0.93	1.56	0.53	0.63	1.52	28.00	0.21

**Visually simple robot/human**								
The happiest	0.80	1.13	2.11	0.39	0.63	1.24	19.00	0.04*
The saddest	0.91	1.27	2.08	0.39	0.93	1.40	23.00	0.09
The most embarrassment thing	1.15	1.80	4.00	0.29	0.47	1.74	16.00	0.02*

Total	1.29	1.57	1.88	0.35	0.65	1.34	15.00	0.02*

*ASD, autism spectrum disorder; TD, typically developing; Q_1_, first quartile; Q_3_, third quartile*.

*Significant at p < 0.05.

In the ASD group alone, significantly more words were used in self-disclosure statements made to the visually simple robot compared to the human interviewer for questions regarding “embarrassing” experiences (*p* < 0.05) and total topics (i.e., embarrassing, happiest, and saddest experiences collectively) (*p* < 0.01). Among ASD participants there were no significant differences in the number of words used in self-disclosure statements directed toward the android robot compared to the human interviewer. In the TD group alone, there were no significant differences in the number of words used in any conversational condition (embarrassing vs. happiest vs. saddest experiences) or across any communicative partner (i.e., human interviewer vs. simple robot vs. android robot). Details are presented in Table 4.

**Table 4:** The number of words in self-disclosure between each robot and human interviewer for adolescents with ASD and TD.

	Robot			Human interviewer				
	Q_1_	Median	Q_3_	Q_1_	Median	Q_3_	*Z*	*p*
**ASD**								

**Android robot**
The happiest thing	7	19	34	5	18	27	−0.24	0.81
The saddest thing	7	13	40	4	15	32	−0.62	0.53
The most embarrassment thing	3	14	38	5	10	28	−0.18	0.86
Total	23	43	124	14	54	63	−0.18	0.86
**Visually simple robot**
The happiest thing	16	27	38	5	18	27	−1.58	0.11
The saddest thing	6	21	36	4	15	32	−1.52	0.13
The most embarrassment thing	9	30	45	5	10	28	−2.40	0.02*
Total	49	85	116	14	54	63	−2.58	<0.01**

**TD**								

**Android robot**
The happiest thing	12.50	23.00	34.25	10.25	23.50	48.50	−0.49	0.62
The saddest thing	8.00	17.50	33.75	6.00	17.50	45.00	−0.98	0.33
The most embarrassment thing	9.75	17.50	22.25	9.25	27.00	54.25	−1.86	0.06
Total	33.50	62.50	83.25	27.50	88.50	144.75	−1.26	0.21
**Visually simple robot**
The happiest thing	13.25	18.00	21.75	10.25	23.50	48.50	−1.54	0.12
The saddest thing	7.00	15.50	23.25	6.00	17.50	45.00	−0.63	0.33
The most embarrassment thing	12.00	17.50	21.75	9.25	27.00	54.25	−1.54	0.12
Total	38.75	55.00	69.25	27.50	88.50	144.75	−1.68	0.09

*ASD, autism spectrum disorder; TD, typically developing; Q_1_, first quartile; Q_3_, third quartile*.

*p < 0.05, **p < 0.01.

We did not find any relationship between IQ and the number of words in self-disclosure to android robot (*r* = 0.20, *p* = 0.41), visually simple robot (*r* = 0.19, *p* = 0.44), and human interviewer (*r* = −0.09, *p* = 0.73) in participants.

## Discussion

Previous studies have suggested that individuals with ASD show preference for certain interactions with robotic systems relative to confederate human interactions (31). In this capacity it has been hypothesized that some individuals with ASD may gravitate toward simple, mechanical objects (32). Hence, in the present study, it was predicted that adolescents with ASD would be expected to show a stronger affinity to visually simple robots, and in our sample ASD adolescents did report higher levels of enjoyment while conversing with the visually simple robot and demonstrated a greater level of self-disclosure with the visually simple robot compared to TD peers.

Adolescents with ASD also reported high levels of enjoyment in conversing with the android robot, but they did not show higher rates of self-disclosure with the android. Previous research showing that individuals with ASD show a strong affinity for robots (33) supports the current study’s observation that the sophisticated technology of the android embodied in ACTROID-F might be reported as favorable by adolescents with ASD. However, in terms of potential for meaningful self-disclosure around personal topics and experiences, it is possible that many adolescents with ASD were so focused or interested in the life-like appearance and movement of the android, that motivation to consider and share personal experiences were lower with the android. Alternatively, as the ACTROID-F is highly human-like in appearance, it is possible that both the android and human interviewer shared similar limitations in eliciting communicative exchanges when compared to the visually simple robot. This may either be due to the comfort levels of the teens with ASD or that the android and the human interviewer created higher levels of sensory stimulation or larger numbers of social cues to manage in comparison to the simplicity of CommU, which was the visually simple robot. Furthermore, it is possible that the limited expressive behavior of the android robot had an effect on self-disclosure by the adolescents.

Aside from length or content of the self-disclosure, the present study does support that interactions with the two robots were positive experience for adolescents with ASD. These results provide preliminary support on the utility of robots to capitalize on engagement and interest of teens with ASD to create a context to work toward improving or practicing skills of conversation, social reciprocity and relationship building.

Spontaneous conversation with another person provides greater insight into the mental states in daily life (34) and is important in fostering and maintaining social relationships. Spoken language through conversation can be a key factor to acquire an understanding of psychological states of oneself and others (35). Perhaps interventions using visually simple robots may assist adolescents with ASD to develop skills in self-awareness and communicating those insights verbally through conversation.

In their guidelines for humanoid robot designs, Ricks and Colton state that individuals with ASD could begin therapy with a simplistic robot, and as comfort levels increase, introduce more realistic human-like robot to evaluate and move toward increased generalization of learned skills (32). The same may be true for skills involving self-disclosure; after adolescents with ASD communicate with visually simple robot over a period of time, the android robot may offer a step toward generation of self-disclosure skills between the visually simple robot and human peer or therapist.

While the current study was not able to, in any way, test generalization or habituation effects, it represents one of the first systematic investigations in self-disclosure using robots for adolescents with ASD. In future work, it would be important to evaluate habituation effects with the two robots by observing interactions over an extended range of time. Second, characteristics of the human interviewer may certainly influence the quality and quantity of self-disclosure provided by the participating adolescents. Our aim was to involve human interviewers matched according to the age and sex of the android (young adults and female). Therefore, we enlisted research assistants working in our laboratory (Caucasian, female, average age: 25 years). Further investigation regarding characteristics of the human interviewer (age, sex, and disposition) might yield interesting results. Third, analyses of the current study were also somewhat limited by the small sample size (*n* = 19) and a larger sample in the ASD and TD groups would be useful for yielding broader and more applicable results, as well as for determining why children with ASD demonstrated lengthier disclosures when interacting with the visually simple robot. Fourth, it is possible that the within-subject design can be prone to the “carryover effect,” which may have affected the results.

Despite limitations, all participants were able to complete study procedures, and results suggest differences of import between ASD and TD teens regarding enjoyment levels in communicating with robots, as well as differences among ASD participants showing higher rates of self-disclosure in interactions with the visually simple robot compared to interactions with a human therapist. As our capacity to utilize technology in intervention and therapeutic settings continues to become a viable option over time, perhaps we can continue to consider ways in which robots represent meaningful contributions to the promotion of conversation, self-awareness, and social engagement with others among those affected by autism.

## Ethics Statement

All procedures involving human participants were conducted in accordance with the ethical standards of the institutional and/or national research committee and with the 1964 Helsinki Declaration and its later amendments or comparable ethical standards.

## Author Contributions

HK designed the study, conducted the experiment, carried out the statistical analyses, analyzed and interpreted data, and drafted the manuscript. ZW, AS, YY, YMA, HT, NS, HI, MM, YMI, and MK conceived of the study and participated in its design and assisted with data collection and scoring of behavioral measures and analyzed and interpreted the data and were involved in drafting the manuscript and revised it critically for important intellectual content. MK was involved in giving final approval of the version to be published. All authors read and approved the final manuscript.

## Conflict of Interest Statement

YY and HI serve as consultants of Vstone Co. Ltd. HI owns stock in the same company. All other authors declare that the research was conducted in the absence of any commercial or financial relationships that could be construed as a potential conflict of interest. The reviewer MM and handling editor declared their shared affiliation.

## References

[1] Losh M Capps L. Narrative ability in high-functioning children with autism or Asperger’s syndrome. J Autism Dev Disord (2003) 33:239–51. 10.1023/A:1024446215446 12908827

[2] Sprecher S Hendrick SS Self-disclosure in intimate relationships: associations with individual and relationship characteristics over time. J Soc Clin Psychol (2004) 23:857–77. 10.1521/jscp.23.6.857.54803

[3] Sprecher S Treger S Wondra JD Hilaire N Wallpe K Taking turns: reciprocal self-disclosure promotes liking in initial interactions. J Exp Soc Psychol (2013) 49:860–6. 10.1016/j.jesp.2013.03.017

[4] Capps L Losh M Thurber C “The frog ate the bug and made his mouth sad”: narrative competence in children with autism. J Abnorm Child Psychol (2000) 28:193–204. 10.1023/A:1005126915631 10834770

[5] Crane L Goddard L Pring L. Brief report: self-defining and everyday autobiographical memories in adults with autism spectrum disorders. J Autism Dev Disord (2010) 40:383–91. 10.1007/s10803-009-0875-4 19777333

[6] Goldman S. Brief report: narratives of personal events in children with autism and developmental language disorders: unshared memories. J Autism Dev Disord (2008) 38:1982–8. 10.1007/s10803-008-0588-0 18512137

[7] Losh M Capps L. Understanding of emotional experience in autism: insights from the personal accounts of high-functioning children with autism. Dev Psychol (2006) 42:809–18. 10.1037/0012-1649.42.5.809 16953688

[8] Barakova EI Gillesen JCC Huskens BE Lourens T End-user programming architecture facilitates the uptake of robots in social therapies. Rob Auton Syst (2013) 61:704–13. 10.1016/j.robot.2012.08.001

[9] Huskens B Verschuur R Gillesen J Didden R Barakova E. Promoting question-asking in school-aged children with autism spectrum disorders: effectiveness of a robot intervention compared to a human-trainer intervention. Dev Neurorehabil (2013) 16:345–56. 10.3109/17518423.2012.739212 23586852

[10] Warren ZE Zheng Z Swanson AR Bekele E Zhang L Crittendon JA Can robotic interaction improve joint attention skills? J Autism Dev Disord (2013) 45:3726–34. 10.1007/s10803-013-1918-4 PMC3949684 24014194

[11] Lee J Obinata G Developing therapeutic robot for children with autism: a study on exploring colour feedback. 2013 8th ACM/IEEE International Conference on Human-Robot Interaction (HRI); Tokyo, Japan: IEEE (2013). p. 173–4.

[12] Lee J Takehashi H Nagai C Obinata G Design of a therapeutic robot for interacting with autistic children through interpersonal touch. 2012 IEEE, RO-MAN; Paris, France: IEEE (2012). p. 712–7.

[13] Wainer J Dautenhahn K Robins B Amirabdollahian F A Pilot study with a novel setup for collaborative play of the humanoid robot KASPAR with children with autism. Int J Soc Robot (2013) 6:45–65. 10.1007/s12369-013-0195-x

[14] Wainer J Ferrari E Dautenhahn K Robins B The effectiveness of using a robotics class to foster collaboration among groups of children with autism in an exploratory study. Pers Ubiquitous Comput (2010) 14:445–55. 10.1007/s00779-009-0266-z

[15] Wainer J Robins B Amirabdollahian F Dautenhahn K Using the humanoid robot KASPAR to autonomously play triadic games and facilitate collaborative play among children with autism. IEEE Trans Auton Ment Dev (2014) 6:183–99. 10.1109/tamd.2014.2303116

[16] Yee AWH Kee TY Limbu DK Jian ATH Dung TA Yuen AWC Developing a robotic platform to play with pre-school autistic children in a classroom environment (2012):81 10.1145/2425296.2425311

[17] Yin T-C Tung F-W Design and evaluation of applying robots to assisting and inducing children with autism in social interaction. Lecture Notes in Computer Science (2013) 8010:524–33. 10.1007/978-3-642-39191-0_57

[18] Diehl JJ Schmitt LM Villano M Crowell CR. The clinical use of robots for individuals with autism spectrum disorders: a critical review. Res Autism Spectr Disord (2012) 6:249–62. 10.1016/j.rasd.2011.05.006 22125579 PMC3223958

[19] Huskens B Palmen A Van der Werff M Lourens T Barakova E Improving collaborative play between children with autism spectrum disorders and their siblings: the effectiveness of a robot-mediated intervention based on Lego^®^ therapy. J Autism Dev Disord (2014) 45:3746–55. 10.1007/s10803-014-2326-0 25428293

[20] Kaboski JR Diehl JJ Beriont J Crowell CR Villano M Wier K Brief report: a Pilot summer robotics camp to reduce social anxiety and improve social/vocational skills in adolescents with ASD. J Autism Dev Disord (2014) 45:3862–9. 10.1007/s10803-014-2153-3 24898910

[21] Zheng Z Young EM Swanson AR Weitlauf AS Warren ZE Sarkar N. Robot-mediated imitation skill training for children with autism. IEEE Trans Neural Syst Rehabil Eng (2016) 24:682–91. 10.1109/tnsre.2015.2475724 26353376 PMC4965236

[22] American Psychiatric Association (APA). Diagnostic and Statistical Manual of Mental Disorders. 5th ed Arlington, VA: American Psychiatric Publishing (2013). p. 5–25.

[23] Lord C Rutter M DiLavore PC Risi S Gotham K Bishop S Autism Diagnostic Observation Schedule, Second Edition (ADOS-2). Torrance, CA: Western Psychological Services (2012).

[24] Rold G Stanford-Binet Intelligence Scales. 5th ed Nelson Education. Rolling Meadows, IL: Riverside (2003).

[25] Rutter M Bailey A Lord C The Social Communication Questionnaire. Los Angeles, CA: Western Psychological Services (2010).

[26] Constantino J Gruber C The Social Responsiveness Scale. Los Angeles: Western Psychological Services (2002).

[27] Nishio S Taura K Sumioka H Ishiguro H Teleoperated android robot as emotion regulation media. Int J Soc Robot (2013) 5:563–73. 10.1007/s12369-013-0201-3

[28] Kumazaki H Muramatsu T Yoshikawa Y Matsumoto Y Miyao M Ishiguro H Tele-operating an android robot to promote the understanding of facial expressions and to increase facial expressivity in individuals with autism spectrum disorder. Am J Psychiatry (2017) 174:904–5. 10.1176/appi.ajp.2017.17030257 28859509

[29] Kumazaki H Warren Z Corbett BA Yoshikawa Y Matsumoto Y Higashida H Android robot-mediated mock job interview sessions for young adults with autism spectrum disorder: a Pilot study. Front Psychiatry (2017) 8:169. 10.3389/fpsyt.2017.00169 28955254 PMC5601082

[30] Shimaya J Yoshikawa Y Matsumoto Y Kumazaki H Ishiguro H Mimura M Advantages of indirect conversation via a desktop humanoid robot: case study on daily life guidance for adolescents with autism spectrum disorders. 2016 25th IEEE, Robot and Human Interactive Communication (RO-MAN); New York, NY, USA: IEEE (2016). p. 831–6.

[31] Robins B Dautenhahn K Dubowski J Does appearance matter in the interaction of children with autism with a humanoid robot? Interact Stud (2006) 7(3):509–42. 10.1075/is.7.3.16rob

[32] Ricks DJ Colton MB Trends and considerations in robot-assisted autism therapy. 2010 IEEE International Conference on Robotics and Automation (ICRA); Anchorage, AK, USA: IEEE (2010). p. 4354–9.

[33] Pierno AC Mari M Lusher D Castiello U. Robotic movement elicits visuomotor priming in children with autism. Neuropsychologia (2008) 46:448–54. 10.1016/j.neuropsychologia.2007.08.020 17920641

[34] Müller E Schuler A. Verbal marking of affect by children with Asperger syndrome and high functioning autism during spontaneous interactions with family members. J Autism Dev Disord (2006) 36:1089–100. 10.1007/s10803-006-0146-6 16897388

[35] Bang J Burns J Nadig A Brief report: conveying subjective experience in conversation: production of mental state terms and personal narratives in individuals with high functioning autism. J Autism Dev Disord (2012) 43:1732–40. 10.1007/s10803-012-1716-4 23179342

