# Supplementary material for: Can Robotic Systems Promote Self-Disclosure in Adolescents with Autism Spectrum Disorder? A Pilot Study
Source: Front Psychiatry. 2018 Feb 9;9:36. doi: 10.3389/fpsyt.2018.00036 (PMC5811466; doi:10.3389/fpsyt.2018.00036)
Supplement: Supplementary file 1 [file data_sheet_1.docx]

***Supplementary Material***

Either of the below three scripts will be used for the conversation with each target agent. Each session will last about 5 minutes.

[Script 1]

Target agent: “Hello, it’s good to see you!” “Please tell me about the happiest thing you ever experienced at home?”

Participant: “....”

Target agent: “OK. Thank you for telling me that. That does seem happy” “Please tell me about the saddest thing you ever experienced at home”

Participant: “…”

Target agent: “OK. Thank you for telling me that. That does seem sad” “Please tell me about the most embarrassing thing you ever experienced at home.”

Participant: “…”

Target agent: “OK. Thank you for telling me that. That does seem embarrassing” “Thank you very much.”

[Script 2]

Target agent: “Hello, it’s good to see you!” “Please tell me about the happiest thing you ever experienced at school”

Participant: “....”

Target agent: “OK. Thank you for telling me that. That does seem happy “Please tell me about the saddest thing you ever experiences at school”

Participant: “…”

Target agent: “OK. Thank you for telling me that. That does seem sad “Please tell me about the most embarrassing thing you ever experienced at school”

Participant: “…”

Target agent: “OK. Thank you for telling me that. That does seem embarrassing. “Thank you very much.”

[Script 3]

Target agent: “Hello, it’s good to see you!” “Please tell me about the happiest thing you ever experienced somewhere outside of school.”

Participant: “....”

Target agent: “OK. Thank you for telling me that. That does seem happy “Please tell me about the saddest thing you ever experienced somewhere outside of school.”

Participant: “....”

Target agent: “OK. Thank you for telling me that. That does seem sad” “Please tell me about the most embarrassing thing you ever experienced somewhere outside of school.”

Participant: “…”

Target agent: “OK. Thank you for telling me that. That does seem embarrassing. “Thank you very much.”

The order of conditions in each participant is as follows:

ASD-1: A-S-H

ASD-2: S-H-A

ASD-3: S-A-H

ASD-4: A-H-S

ASD-5: H-S-A

ASD-6: A-S-H

ASD-7: H-A-S

ASD-8: S-H-A

ASD-9: A-H-S

ASD-10: H-A-S

ASD-11: H-S-A

TD-1: A-S-H

TD-2: S-H-A

TD-3: H-A-S

TD-4: A-H-S

TD-5: H-S-A

TD-6: S-H-A

TD-7: A-S-H

TD-8: S-A-H

*Notes: A = android robot, S = visually simple robot, H = human interviewer*
